# Central Modulation of Neuroinflammation by Neuropeptides and Energy-Sensing Hormones during Obesity

**DOI:** 10.1155/2017/7949582

**Published:** 2017-08-23

**Authors:** Roger Maldonado-Ruiz, Lizeth Fuentes-Mera, Alberto Camacho

**Affiliations:** ^1^Laboratory of Virology and Immunology, Faculty of Life Sciences, Autonomous University of Nuevo Leon, San Nicolás de los Garza, NL, Mexico; ^2^Neurometabolism Unit, Center for Research and Development in Health Sciences, Autonomous University of Nuevo Leon, San Nicolás de los Garza, NL, Mexico; ^3^Biochemistry Department, Faculty of Medicine, Autonomous University of Nuevo Leon, San Nicolás de los Garza, NL, Mexico

## Abstract

Central nervous system (CNS) senses energy homeostasis by integrating both peripheral and autonomic signals and responding to them by neurotransmitters and neuropeptides release. Although it is previously considered an immunologically privileged organ, we now know that this is not so. Cells belonging to the immune system, such as B and T lymphocytes, can be recruited into the CNS to face damage or infection, in addition to possessing resident immunological cells, called microglia. In this way, positive energy balance during obesity promotes an inflammatory state in the CNS. Saturated fatty acids from the diet have been pointed out as powerful candidates to trigger immune response in peripheral system and in the CNS. However, how central immunity communicates to peripheral immune response remains to be clarified. Recently there has been a great interest in the neuropeptides, POMC derived peptides, ghrelin, and leptin, due to their capacity to suppress or induce inflammatory responses in the brain, respectively. These may be potential candidates to treat different pathologies associated with autoimmunity and inflammation. In this review, we will discuss the role of lipotoxicity associated with positive energy balance during obesity in proinflammatory response in microglia, B and T lymphocytes, and its modulation by neuropeptides.

## 1. Introduction

The first line of defense of an organism before any invasion of pathogens or tissue damage is the innate immune system. It includes physical barriers such as the skin, or specific cell types such as macrophages and complement proteins; as a whole, it modulates the inflammatory response. The inflammatory response consists of an innate cellular system and humoral responses that occur during injury, such as exposure to cold or heat, ischemia, and trauma. The inflammatory response can be divided into two types, depending on the cell type and intensity-duration of the stimulus: (1) acute inflammation, characterized by a time window of minutes to hours and by the abundant presence of neutrophils; (2) chronic inflammation, which in time extends from days up to years, and accumulation of lymphocytes in the inflamed tissue predominates. In this context, precise activation of the inflammatory response is coordinated by the involvement of various cell types including recruitment of macrophages and leukocytes, activation of endothelial cells, platelet aggregation, and release of various cytokines including interleukin 1 (IL-1), interleukin 6 (IL-6), and tumor necrosis factor alpha (TNF-*α*). It is through these events that the body physiologically restores the cellular homeostasis and defends the organism from external injuries [1]. However, despite the sophisticated modulation of the inflammatory response in time and space, the chronic release of inflammatory signals promotes the development of diseases such as cancer, hypertension, cardiovascular disorders, and metabolic disorders including diabetes and obesity.

The link between the immune system and the regulation of body energy metabolism has started to be understood in the recent years. Initial studies identified selective cellular types for the immune system including pulmonary alveolar macrophages, peritoneal exudate monocytes, and polymorphonuclear leukocytes, which delegate their energy requirement to specific metabolic pathways, depending on the tissue in which they reside. For example, macrophages activate oxidative phosphorylation, whereas monocyte and polymorphonuclear leukocytes are mainly glycolytic [2]. In addition, during an inflammatory event, macrophages increase the catalysis of metabolic enzymes such as hexokinase and citrate synthase in addition to high glucose consumption [3], suggesting an increase in the glycolysis rate during phagocytosis or secretory activity. These studies established the immune system-metabolism relationship in a cellular process called “immunometabolism” [4]. Recently, research has shown that metabolic regulation not only depends on the activation of specific metabolic pathways in a cell type, but that the immune system regulates body metabolism and plays an important role in the development of metabolic disorders such as metabolic syndrome and obesity. Obesity has been characterized as an atypical form of inflammation induced primarily by the accumulation of fatty acids in tissues, altering the metabolic regulation, including liver, adipose tissue, and muscle. This type of inflammation was termed “metainflammation” or “metabolic inflammation” [5].

Positive energy balance during maternal overnutrition or obesity lead to changes in plasma and tissue specific lipidomic profile that might promote inflammation. In fact, saturated lipids have been shown to represent a group of molecules as more active candidates in promoting inflammation through their interaction with toll-like receptors TLR1 and TLR4 and by activating nuclear factor kappa B (NF-*κ*B), a promoter of inflammatory genes [6, 7]. This type of inflammation is not limited to peripheral tissues; it extends to much more distant borders and is able to reach the CNS, promoting the development of neuroinflammation [5]. However, it is also possible that resident brain cells, such as microglia, may induce neuroinflammation independently of their peripheral activation [8]. Paradoxically, modulation of the cytokine-dependent inflammatory signal is controlled by the activation of antagonist cytokines such as IL-10, TGF-*β*, IL-11, and agonist receptor IL-1, among other cytokines and interleukin soluble receptors. These cytokines function as anti-inflammatory, inhibiting the activation of macrophages, T lymphocytes, and natural cytotoxic cells (NK) [1]. Recent studies have demonstrated the involvement of molecules produced in the CNS in the regulation of inflammatory and energetic metabolism, proposing that neuropeptides are synthesized by macrophages, lymphocytes, and neutrophils to regulate inflammation and metabolism [9, 10]. In this review, we will describe the signaling pathways involved in the process of metabolic inflammation in a scenario of positive energy balance and its modulation by neuropeptides.

## 2. Lipotoxicity Is a Mediator of Metabolic Inflammation in the CNS

Epidemiological data confirms a strong link between the increase in the level of obesity and the development of type 2 diabetes, indicating that for every kilogram of gained weight, at the population level, there is a linear increase in the diabetes rate [11]. Experimental evidence, in obese humans and animal models with obesity, suggests that the leakage of lipids from adipose tissue and ectopic accumulation of ceramides (a type of sphingolipid), acylcarnitines, diacylglycerols, and saturated fatty acids cause tissue damage to metabolically relevant organs, including the skeletal muscle, liver, pancreatic beta cells, myocardium, and brain, in an event called lipotoxicity [12–14]. The lipotoxic effect is largely determined because every organ has its own lipid profile. Hence, selective changes in lipid species in different organs may be relevant to the development of lipotoxicity. In this context, it is known that, physiologically, C18:0 type ceramides are essential for cerebellar development and C22:0 and C24:0 ceramides regulate hepatic function [12, 13], while saturated diacylglycerols and lipids take part in intracellular signaling processes in many cellular types of the body [14]. In this regard, it has been suggested that, during obesity, new lipid species, which are potentially toxic for the body's organs, are produced, including ceramides, cholesterol, saturated fatty acids, and diacylglycerols. All these species are known to inhibit insulin sensitivity in cellular cultures and animal models [15]. Saturated ceramide and lipid accumulation has even been detected in the skeletal muscle of obese humans, which correlates to insulin resistance [15]. Recent evidence has shown substantial association between lipidomic profile leading to lipotoxicity and activation of neuroinflammation.

Previously, the brain was considered an immunologically privileged organ, partly because the lack of constitutive expression of MHC class I and class II and the absence of classical antigen-presenting cells (APCs) and lymphatic vessels. However, the identification of peripheral immune system cells including B cells and T lymphocytes in genetic and nutritional models of obesity has proposed that the metabolic inflammation observed during obesity is able to colonize the CNS and promote central inflammation such as microglia activation [1]. Each of these cell populations will promote an inflammatory state through the secretion of antibodies and interleukins [16, 17]. We will now describe some of the cell populations that have been implicated in the process of neuroinflammation activation in a lipotoxic context during obesity.

### 2.1. Microglia

Microglia represent a selective cell type with characteristics of CNS resident macrophages, which originate from erythromyeloid progenitors derived from yolk sac cells during the embryonic stage and which subsequently colonize the brain during embryonic development [18, 19]. Physiologically, their activation is required for the proper functioning of the CNS as they positively modulate neurogenesis and synaptic plasticity in addition to acting as major APCs in the CNS [20].

The relationship between lipotoxic damage in the context of obesity and the activation of central inflammation is based on evidence showing that the exposure of high fat diet in rodents promotes inflammation in the CNS that culminates as damage to the regions of the hypothalamus, cognitive deterioration, and decreased neurogenesis [20, 21]. Molecularly speaking, it is proposed that, similar to macrophages that regulate innate inflammatory activation in the peripheral system in a lipotoxic context, the activation of microglia in the brain is induced by the interaction of fatty acids with TLRs. In fact, lauric acid (C12:0) and palmitic acid (C16:0) lipids have been identified as inducing TLRs migration to lipid rafts, heterodimerization of TLR1 and TLR2 receptors, and the homodimerization of the TLR4 in macrophages [22, 23]. In addition, experimental evidence has identified the recruitment of the MyD88 protein and NADPH to these domains correlating with the production of reactive oxygen species (ROS) [22–24]. Our research group has shown that the stimulation of neurons with palmitic acid recruits the inflammatory related serine/threonine-protein kinase TANK-binding kinase 1 (TBK1) to lipid raft domains, which correlates with insulin resistance [25]. These experiments demonstrate that activation of TLR4 participates in the secretion of inflammatory cytokines via the IKK-NF-*κ*B pathway in microglia, inducing alterations in the hypothalamus and other regions of the CNS [17, 21, 26], and potentially the recruitment of monocytes from the periphery dependent on the increase of TNF-*α* [27]. Taken together, exposure of saturated fatty acids favors the interaction and dimerization of TLR1, TLR2, and TLR 4 towards the lipid rafts microdomain, recruitment of NADPH oxidase and MyD88, and production of ROS, where it will be activated in parallel NF-*κ*B by the IKK and possibly TBK1. Thus, NF-*κ*B-dependent transcriptional activation will promote the secretion of inflammatory cytokines TNF-*α*, IL-*β*1, and IL-6 and altered metabolic profile, as we have recently proposed [28].

### 2.2. T Lymphocytes

The role of T lymphocytes as mediators of metabolic inflammation was initially reported in 2009. Exposure of mice to high fat diet promotes the infiltration of CD8^+^ T lymphocytes into adipose tissue favoring the infiltration of M1 macrophages with inflammatory profile and generating insulin resistance, while its inactivation by specific antibodies represses this phenomenon [29]. Inflammatory activation in the adipose tissue of obese mice is potentiated by two possible scenarios: (1) by reducing expression of the transcriptional factor Foxp3 in T_reg_ lymphocytes [30], cells responsible for regulating the inflammatory response and suppressing autoimmune reactions [31] and (2) by activating a proinflammatory subtype of CD4^+^ T helper cells called Th1 [32]. Activation of these molecular pathways have been widely identified in the generation of insulin resistance and type 2 diabetes mellitus in obese subjects. In fact, it has been proposed that the activation of T lymphocytes in adipose tissue is a key event and depends on the presentation of antigens by MHC class II in CD4^+^ T cells and a costimulatory signal [27]. This mechanism has been described to promote the synthesis of IL-2, where additional interaction of TRC-MHCII is required for the activation of the coreceptor CD28. Also, the TRC-MHCII interaction recruits the Zap70 protein to the CD3 coreceptor allowing the activation of the PLC*γ*-PIP2-PKC*θ* cascade, downstream activation of ERK, and c-Fos expression. On the other hand, the CD28 coreceptor via the PI3K pathway activates MEKK and JNK allowing the production of c-jun. Both TCR and CD28 lead to the transcriptional factor AP-1 nuclear translocation, inducing the expression of IL-2 [31]. In addition, the TCR-CD28 binomial in CD4^+^ T lymphocytes promotes PKC*θ* to activate the CARMA1-Bcl10-MALT1 complex by inducing the activation of NF-*κ*B and TBK1 and the proliferation, differentiation, and production of IL-2, dependent on AP-1 [33, 34]. Thus, in a lipotoxic context, we might suggest that the interaction of CD4^+^ T lymphocytes with an antigen-presenting cell would allow differentiation towards the Th1 subtype by altering the Th1/T_reg_ ratio towards proinflammatory, interferon-producing (INF-*γ*) T cells, IL-2, and TNF-*α* and decreased IL-10 production activity of T_reg_ cells. All this would allow the polarization of macrophages to the M1 phenotype producing proinflammatory cytokines TNF-*β*, IL-1*β*, and IL-6. On the other hand, TCR-MHCI interaction by CD8^+^ T lymphocytes could secrete MCP1 exacerbating the recruitment of macrophages to adipose tissue and increasing inflammation.

**Figure 1:**
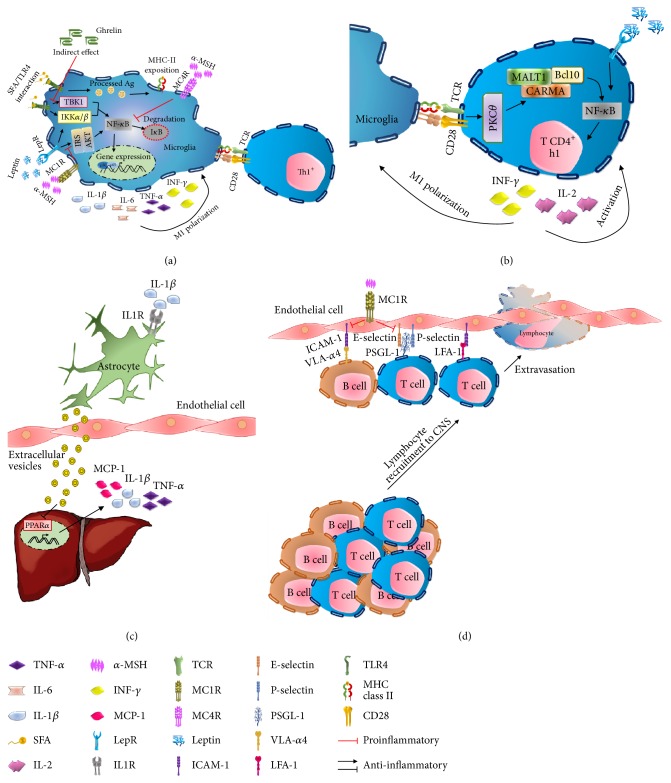
*Immunomodulatory mechanism exerted by neuropeptides in microglia exposed to a lipotoxic stimuli*. (a)* Microglia pro- and anti-inflammatory stimuli*. In microglia, fatty acids and leptin can induce cytokine secretion through TLR4/IKK/NF-*κ*B pathway, but only leptin can activate NF-*κ*B through LepR/IRS1/AKT pathway. Also, leptin induces MHC class II expression leading to T lymphocytes activation. Cytokines have paracrine and autocrine effects. *α*-MSH inhibits the I*κ*B degradation through MC4R and by blocking the LepR pathway through MC1R. Ghrelin blocks the TLR4/IKK/NF-*κ*B pathway activation in microglia cells by indirect effects. (b)* T lymphocyte activation*. Microglia presents the antigen to CD4+ T cells and through the receptor complex MHCII/B7-TCR/CD28 these cells proliferate to the proinflammatory phenotype Th1 which produce IL-2 and INF-*γ* through PKC*θ*-CARMA-MALT1-Bcl10/NF-*κ*B complex and by leptin action. (c)* Astrocytes inflammatory mechanism*. IL-1*β* induces the secretion of extracellular vesicles which inhibits PPAR*α* expression on hepatocytes leading to TNF-*α*, IL-1*β*, and MCP-1 production facilitating lymphocyte infiltration to CNS. (d)* Lymphocyte extravasation to CNS*. Inflammatory signals such as cytokines and CMP-1 promote the expression of adhesion proteins E-selectin, P-selectin, and ICAM-1. Lymphocytes can interact with the adhesion proteins through its own integral proteins VLA-*α*4, PSGL-1, and LFA-1 and cross the BBB. Melanocortins prevent T-cell infiltration by the *α*-MSH-MC1R interaction which blocks externalization of adhesion proteins.

Activation and recruitment of lymphocytes to adipose tissue during positive energy balance in obesity also cause them to migrate to more distant borders and interact with CNS cells, including microglia, as described for various pathologies such as experimental autoimmune encephalomyelitis and cerebral ischemia [33, 35]. In a summarized way they involve the attraction of T cells to the site inflamed by chemokine such as Interferon-Inducible T-Cell Alpha-Chemoattractant (I-TAC), interferon gamma-induced protein 10 (IP-10), and monokine induced by gamma interferon (MIG) [36], expression of the E and P selectins in endothelial cells that serve as anchor for their ligands in T lymphocytes, PSGL-1, and *α*4-integrin, facilitating the transport of lymphocytes through blood vessels (Figure 1). Finally, the ultimate barrier for the invasion of T lymphocytes into the nervous system is represented by the blood-brain barrier (BBB), which, by expressing the LFA-1 membrane protein in T cells, can bind to the ICAM-1 protein endothelial cells and cross the BBB leaving morphologically intact narrow junctions. This has been corroborated in recent studies, showing the inhibition of the expression of these adhesion proteins, reducing the infiltration of T lymphocytes into the brain during an inflammatory event [37, 38], which is presumably regulated by poly(ADP-ribose) polymerase-1 (PARP) [38]. Despite the evidence supporting the infiltration of T lymphocytes into the nervous system, molecular and cellular mediators that mediated the communication between the brain and the immune system remained unidentified. Maybe the first evidence to support CNS-peripheral immune system cross-talk was recently identified by showing that selective inflammatory stimulus (IL-1*β*) into the CNS and astrocytes secrete extracellular vesicles (EV), which cross the BBB and reach organs such as the liver allowing the suppression of PPAR*α* and favoring the production of TNF-*α* and IL-*β*1 and monocyte chemoattractant protein-1 (MCP-1). Cytokines production promotes the T lymphocytes recruitment into the inflamed brain region [39] (Figure 1).

### 2.3. B Lymphocytes

B lymphocytes represent a cell type of the immune system, originating from hematopoietic cells that mature in bone marrow and participate in innate and adaptive immunity. Their main function is the production of antibodies against foreign antigens into the body [40]. In addition, they also function as APCs by presenting the antigens to T lymphocytes that were initially captured by their B-cell receptor and promote inflammation by secreting proinflammatory cytokines such as IL-6 and TNF-*α*, thus favoring the polarization of T cells into a proinflammatory phenotype [40]. The molecular mechanisms that promote the migration of B lymphocytes to the CNS are not yet fully understood; however, it has been proposed that its association with membrane proteins, such as the *α*-4 subunit of VLA-A, ICAM-1, and ALCAM, allows passage through the BBB [41, 42].

Unlike the role of macrophages and Th1 lymphocytes in the modulation of metabolic inflammation during obesity, the impact of B lymphocytes in this context has not yet been fully understood [43]. However, there are several reports that justify its presence and potential participation in the modulation of inflammation at the CNS level. In the first instance, B lymphocytes possess TLR capable of responding to microbial antigens in a T lymphocyte-dependent manner [44], which have been identified to actively mediate metabolic inflammation by interacting with fatty acids [23, 26]. In fact, the accumulation of antibodies of the IgG class in the microglia of the ARC nucleus has been observed in mice exposed to a hypercaloric diet, polarizing it towards the M1 phenotype through its Fc receptor [45]. B lymphocytes itself from obese mice might produce a proinflammatory IgGc class which, when administered to mice deficient in B cells, increases the production of proinflammatory cytokines, the polarization of M1 macrophages, and the activation of T lymphocytes [16]. These studies confirm that the function of B lymphocytes in a metabolic compromise scenario seems to be deleterious and promotes metabolic inflammation, leading to the belief that its inhibition could prevent this mechanism. Experimental data demonstrate that this hypothesis is partially true, since the elimination of B lymphocytes using a CD20-specific antibody in a murine model of obesity induced by high fat diet improved glucose tolerance, reduced insulin levels, and reduced the inflammatory profile in adipose tissue. However, the total elimination of B and T lymphocyte populations has no effect [46]. In this scenario, defects in B-cell function have been reported in situations of metabolic compromise as presented in diabetic and nondiabetic obese patients. Subjects with this metabolic profile show a low response to antibodies and secrete a greater amount of IL-6 and TNF-*α* than healthy subjects, and only obese and diabetic patients have a decrease in the production of IL-10, a key cytokine in the suppression of the immune response mediated by B cells [37, 47]. In this way, it is possible that, in a lipotoxic context, the interaction of B lymphocytes with fatty acids or their recruitment to the CNS by glial cells [48] plays a key role in metabolic inflammation, through secretion of inflammatory cytokines, the activation of CD4^+^ T lymphocytes, and microglia polarization towards the M1 phenotype, through MHC class II mediated antigen presentation and the Fc fraction of the antibodies, respectively (Figure 1).

## 3. Energy-Sensing Hormones and Neuropeptides Modulate Central Inflammation 

Neuropeptides are small molecules composed of amino acids, produced mainly but not exclusively by cells of the nervous system, and regulate important physiological processes, including reproduction, feeding, regulation of body weight, memory, anxiety, mood, excitement, reward, and sleep/wake stages [49]. Anti-inflammatory properties of various neuropeptides have been identified in the context of positive energy balance, which include alpha-melanocyte-stimulating hormone (MSH-*α*), vasoactive intestinal peptide (VIP), and neuropeptide Y (NPY) [10, 50, 51]. There is also evidence of the involvement of hormonal signals dependent on ghrelin and leptin on the modulation of an anti-inflammatory phenotype in microglia [49, 52]. In the next section, we will describe evidence of the involvement of peptides derived from prohormone proopiomelanocortin (POMC) and the ghrelin and leptin hormones as potential central modulators of microglia-dependent inflammation in a context of positive energy balance.

### 3.1. Peptides Derived from POMC

Melanocortins are posttranslational products of the POMC gene which is expressed in the arcuate nucleus (Arc) of the hypothalamus, from which a family of opioids and melanocortins products are synthetized including *β*-endorphin, adrenocorticotropic hormone (ACTH), and the melanocyte stimulating *α*, *β*, and *γ* hormones (MSH). This neuropeptide system is unique since its regulation depends on two small endogenous proteins, the peptide-like agouti and the Y neuropeptide [53, 54].

The ability of melanocortins as anti-inflammatory agents is well documented in different models of peripheral inflammation [55]. Melanocortins exert their action through their interaction with the MC1R receptor located in immune cells innate neutrophils, macrophages, and dendritic cells also in microglia, in addition to possessing a high affinity towards MSH-*α*. The administration of MSH-*α* has shown to reduce the production of IL-1, IL-6, and TNF-*α*, and monocyte receptor expression is upregulated in the presence of stimuli such as lipopolysaccharides (LPS) or cytokines. At the CNS, systemic administration of MSH-*α* has been reported to reduce cytokine expression during cerebral ischemia and decrease inflammation at the hippocampal level by inhibiting LPS or IL-1*β* induced dinoprostone (PGE2) secretion. MSH-*α* also reduces the production of nitric oxide (NO) and prostaglandin (PG) favored by IL-1*β* in the hypothalamus of rats [56]. In the past decade, MC3R and MC4R receptors have been proposed as responsible for the anti-inflammatory action of melanocortins in the brain. This proposal is based on studies demonstrating that the expression of these two receptors is higher in comparison to the other members of this group [57] and that the administration of MSH-*α* reduces the hypothalamic production of iNOS and COX2 in rats administered with LPS and decreases the expression of TNF-*α* induced by LPS and INF-*γ* in neurons expressing the MC4R, whose effect is blocked by the administration of the MC4R antagonist [58].

Scientific evidence suggests that melanocortins exert their anti-inflammatory activity by inhibiting the transcription factor NF-*κ*B [59] and by inducing IL-10 in microglia through the MC4R receptor. In astrocytes, both brain-derived neurotrophic factor (BDNF) and peroxisome-proliferator-activated receptor gamma (PPAR*γ*) expression have been observed to be regulated by the MC4R-cAMP-PKA-CREB pathway [60]. On the other hand, activation of the MC1R receptor using the pharmacological agonists MS05 and MS09 is able to reduce the expression of E-selectin and VCAM, in addition to reducing the activation of NF-*κ*B in endothelial cells exposed to TNF-*α*. Knowing that E-selectin and VCAM represent integral membrane proteins important for the migration of B and T lymphocytes towards the site of inflammation [41], it is proposed that the blockade of the extravasation of these cells represents an anti-inflammatory mechanism parallel to that described by melanocortins. Finally, there are reports that have shown that activation of the MC1R receptor represses the leptin-dependent inflammation in a lipotoxic context [61, 62]. Thus, it is tentative to propose that, in a lipotoxic scenario, melanocortins block the inflammatory process by four main events: (1) increase the secretion of IL-10 from the microglia, (2) decrease the activation of NF-*κ*B, (3) block the action proinflammatory effects of leptin, and (4) prevent infiltration of lymphocytes through the BBB to the CNS.

### 3.2. Ghrelin

It is a peptide of 28 amino acids secreted mainly by the stomach and duodenum, although it is also produced by neurons in the arcuate nucleus [63]. However, overproduction of ghrelin in the hypothalamus promotes food consumption and increases body weight [64]. Ghrelin seems to induce acute peripheral insulin resistance independent of growth hormone (GH), cortisol, and basal serum free fatty acids [65] and both insulin and ghrelin exert regulatory effects on each other [66, 67]. Two types of ghrelin, des-acyl-ghrelin (DAG) and acyl-ghrelin (AG), are known to regulate food intake and growth hormone secretion and influence glucose homeostasis, neuroprotection, memory, immunity, and neuroinflammation [63, 68]. Its main function is to act as an orexigenic signal by antagonizing the effects of leptin via the NPY/Y_1_R axis, through its interaction with the growth hormone secretagogues receptor (GHSR) in NPY and AgRP neurons.

Obesity is known to promote an imbalance in the hormonal profile of obese individuals. Changes in the AG/DAG ratio in obese and metabolic abnormal Italian children compared with normal weight children have been reported [69]. The authors found a 81% increase in AG in obese and metabolic abnormal children when compared with healthy children [69]. Also, recently it has been documented that AG concentrations are higher in plasma of obese patients (435 pg/mL) than nonobese patients (167 pg/mL) [70]. Although it has been stated that during obesity ghrelin plasma levels are decreased in obese individuals as a compensatory mechanism to reduce appetite [71, 72], it only refers to total ghrelin in plasma, given that AG depicts 10% of total ghrelin. In this context, decreasing levels of this hormone may be potentially related to a decrease in DAG concentration [70]. In fact, diet induced obesity (DIO) in mice by high fat diet exposure leads to 15% increase in preproghrelin mRNA-producing cells than control [73]. In addition, both DIO and ob/ob mice model had normal plasma levels of ghrelin which correlates with a decrease in DAG plasma levels [73]. It is known that DAG is metabolized to AG by action of the ghrelin O-acyltransferase (GOAT); not only does the importance of this enzyme lie in its ability to acetylate the unacetylated form of ghrelin, but it has been reported that knocking down the GOAT gene protects mice from obesity induced diet, improves insulin sensitivity, and reduces adiposity when fed HFD and high glucose diet [74]. Furthermore, there is a positive correlation between body mass index (BMI) and GOAT concentration in obese patients, where BMI > 50 had increased concentrations (+34%) compared with normal weight controls [75]. These evidences suggest that an alteration in the AG/DAG ratio related to GOAT activity is potentially important to contribute to metabolic alterations observed during obesity and diabetes. This proposal is tested in recent reports showing that decreasing AG plasma levels associates with positive effects in metabolic disorders, such as decreasing postprandial glucose levels and improvement of insulin sensitivity in overweight patients with type 2 diabetes [76, 77].

On the other hand, the anti-inflammatory and neuroprotective properties of ghrelin have been demonstrated in experimental cord injury (SCI) models, where the administration of ghrelin inhibits the activation of the p38 AMPK/NF-*κ*B pathway followed by the release of the factor of nerve growth (proNGF). These data were corroborated in in vitro models showing that ghrelin stimulation prevented the activation of the AMPK and JUN signaling pathway in addition to reducing the production of ROS in microglia stimulated with LPS [78]. Other studies demonstrated that the intracerebroventricular administration of ghrelin reduces the mRNA expression of the proinflammatory cytokines IL-1*β*, IL-6, TNF-*α*, INF-*γ*, and iNOS in the blood of rats subjected to a 70% calorie restriction by one week [79]. However, it appears that this mechanism is independent of the GHSR1*α* receptor, since this receptor is not expressed in the resident microglia of the brain and spinal cord or in primary culture. In this context, ghrelin might potentially act by blocking the expression of the matrix metalloproteinase 3 (MMP-3) on dopaminergic stressed cells [80]. In addition to this, ghrelin has been proposed as a neuroprotective agent by decreasing the production of proinflammatory cytokines, IL-1*β*, IL-6, TNF-*α*, iNOS, and ROS, by microglia in models of amyotrophic lateral sclerosis, neurotoxicity, neuronal death induced by kainic acid, experimental autoimmune encephalomyelitis, Parkinson's, and Alzheimer's [49, 81]. In addition, it blocks the activation of the microglia and reduces infiltration of T lymphocytes towards the spinal cord against a challenge with LPS [78, 82]. Ghrelin also prevents the differentiation of a proinflammatory T-cell subtype, termed Th17, by blocking the activation of the mTOR/STAT3 pathway [83]. In relation to diseases closely related to metabolism, ghrelin has been linked to attenuation in the activation of the TLR4/MyD88/TRAF6/NF-*κ*B pathway and cell death in pheochromocytoma cells (PC12) in a model of diabetic encephalopathy [84]. Taken together, these data allow us to hypothesize that ghrelin plays an important role in the regulation of metabolic inflammation in the CNS by modulating the secretion of proinflammatory cytokines by microglia.

### 3.3. Leptin

**Figure 2:**
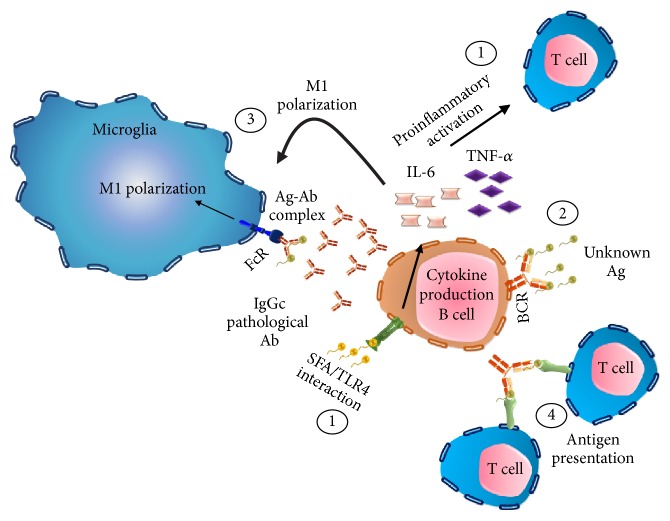
*B lymphocytes regulate neuroinflammation*. (1) Saturated fatty acids induce the secretion of proinflammatory cytokine through the interaction with the TLR4 located in B lymphocytes, favoring the polarization of lymphocytes and microglia activation to a proinflammatory phenotype. (2) Production of a pathogenic IgG class antibody (Ab) regulated by an unknown antigen (Ag). (3) Microglia M1 polarization trough the Ab-Fc receptor interaction. (4) B cells receptor (BCR) mediated antigen presentation.

Leptin is a peptide hormone with a molecular structure similar to interleukins, composed of 146 amino acids, its synthesis is primarily in adipocytes, and it travels through the circulation to reach the CNS, where it interacts with the leptin receptor (LepR) located in hypothalamic neurons and regulates food intake through an anorexigenic signal [85]. It is also involved in hematopoiesis, angiogenesis, and glucose metabolism and has a proven role in cells innate and adaptive immune system [85]. In normal human patients, leptin plasma levels are around 21.6 pg/mL, whereas obese patients show higher concentrations (61.9 pg/mL) [70] Experimental data show that the LepR has the ability to activate signaling pathways associated with inflammatory profiles including those of IL-6, the JAK-STAT, and MAPK-PI3K pathway, regulates the production of IL-2 and INF-*γ* in Th1 lymphocytes, and reduces the production of anti-inflammatory IL-10 cytokine [86]. Furthermore, it appears that leptin and swelling ratio is positive feedback type as systemic injection of LPS to mice increases the concentration of mRNA in adipose tissue leptin mice and induces the secretion of IL-1*β*; besides TNF-*α* and IL-1 cytokines regulate the expression of leptin. Additionally, the inflammatory effect exerted by leptin is dependent on modulation at the level of adhesion molecules expression such as ICAM-1 and VLA2 in CD4^+^ cells, preventing proliferation of suppressor cells of the immune response type T_reg_ Foxp3 [31, 87]. The role of this hormone on the proinflammatory action of B lymphocytes seems limited to increase phosphorylation STAT3, a crucial mechanism in the production of TNF-*α*, accompanied by low phosphorylation in AMPK, crucial for the activation of E47 through phosphorylation of p38 AMPK; and inhibiting apoptosis in a mice model exposed to HFD [88]. The proinflammatory effects of leptin on microglia are by nature proinflammatory and induce secretion of IL-1*β* cytokine-dependent stimulation with LPS, by a mechanism independent caspase 1, IL-6 by the pathway IRS1-PI3K-AKT-NF-kB and TNF-*α*, and CINC-1 MIP-2 chemokines [89]. In fact, IL-6 has proved to have a sensitizing action to leptin in hypothalamic neurons in obese animals by exposure to high fat diet [90]. It has been observed that in microglia deficient mouse leptin (ob/ob) there is a downregulation in genes integrin-alpha X (Iigax), NALP3, and molecule F4/80, important for correct development of the inflammatory response and T-cell differentiation regulated by APC [91] (Figure 2).

## 4. Potential Treatments for Inflammation in Metabolic Related-Diseases

Obesity is a worldwide health problem showing failure in pharmacologic and therapeutic interventions to ameliorate its metabolic complications. Diet might show the first potential avenue to modulate this pandemic. It is widely reported that polyunsaturated fatty acids (PUFAs) had beneficial effects on several metabolic related-diseases, such as obesity. For instance, omega-3 fatty acids (*ω*-3 PUFAs) inhibit mammary tumor progression in obese mice [92], like wise this fatty acids has anti-inflammatory effects in the adipose tissue and hypothalamus [93, 94], and protect against insulin resistance and dyslipidemia by suppressing the activation of TLR4 [95, 96]. The key receptors involve in this beneficial effects are the G protein-coupled receptor (GRP), specifically the selective GRP40 and GRP120, which has been proposed has possible therapeutic targets for insulin resistance and metabolic inflammation [97, 98]. In fact, administration of a GRP40 agonist (Yhhu4488) promotes high expression of glucagon-like peptide-1 (GLP-1), decreased fasting blood glucose level, improved *β*-cell function and lipid homeostasis in type 2 diabetic ob/ob mice [99]. Also, GRP120 stimulation by a selective agonist improved glucose tolerance, decreased hyperinsulinemia, increased insulin sensitivity and decreased hepatic steatosis in a DIO mice model [100]. Of note, the role of GRP40 and GRP120 might be potentially relevant given that both show high expression within the hypothalamus and the combined activation of both receptors results in better metabolic outcomes [98].

Another possible neuroinmmunometabolic target are the kinases TBK1 and IKK*ε* related to inflammatory pathways. These proteins had been reported to participated in the development of insulin resistance and diabetes [25, 101]. In addition, we proposed recently that TBK1 may have a significant role in the microglia-mediated neuroinflammation observe during obesity [28]. In fact, it has been demonstrated that the administration of an specific TBK1-IKK*ε* inhibitor (amlexanox), reduces weight, insulin resistance, fatty liver and inflammation, as well as increased energy expenditure [102]. Together, these data suggest that dual-specificity inhibitors of IKKɛ and TBK1 may be effective therapies for metabolic disease in an identifiable subset of human patients [103].

Finally, the ghrelin system might represent another possible molecular target for immunomodulation. The administration of des-acyl-ghrelin analog (AZP531) prevent dysregulation of glucose homeostasis in C57BL/6J mice exposed to a HFD [104]. Furthermore, chronic exposure to an inhibitor of AG secretion (CF801) decreased weight gain and adiposity without affecting caloric intake [105]. More recently, a synthetic triterpenoids has been proposed as a potential therapeutic agent to treat diabetes and obesity., due to its ability to inhibit ghrelin acylation by the human isoform of GOAT (hGOAT), these compounds function as covalent reversible inhibitors of hGOAT [106]. Thus, blocking proinflammatory signals through GRPs or nuclear factors inhibitor such as TBK1, and reducing AG plasma levels, might be potential pharmacologic treatments to obesity and metabolic disorders.

## 5. Conclusions

We contemplate that the activation inflammation associated central lipotoxicity in a scenario of positive energy balance is dependent on time and intensity of the stimulus. At early times of ingestion of a high fat diet, lipids interact with toll-like receptors (SFA-TLR4) activating inflammatory pathway MyD88/IKK/NF-*κ*B and possibly the TBK1 protein, initiating secretion of proinflammatory cytokines IL-1*β*, IL-6, TNF-*α* and INF-*γ*. Meanwhile, in a parallel scenario, increased inflammation at the level of adipose tissue promotes increased concentration of leptin in the plasma and by promoting the expression of adhesion proteins on the cells of the BBB, as ICAM-1, VLA-2 and ALCAM, cells might recruit peripheral immune into the CNS by the action of IL-1*β*. In particular, leptin increase might sensitize microglia subsequent to proinflammatory stimuli and will induce expression of MHC class II and expression of IL-1*β*. At late, cells such as B and T lymphocytes and macrophages could infiltrate the CNS, where microglia would serve as an APC cell to T-cell and by TCR-CD28/MHCII interaction, might recruit the complex CARMA1-Bcl10 -MALT1, allowing activation NF-kB and IL-2 secretion. In this state T cells could be placed in a state of Th1 type secreting cell or inflammatory cytokines. Likewise, a reduction would be expected in the production of IL-10 because of the action of the INF-*γ* and SFA on T_reg_ cells. B cells attracted to the CNS begin producing IgG toxic antibodies that will accumulate within microglia and will be able to act as ACP with T cells. This intricate network of cells and cytokines form a positive feedback loop that amplifies the effect initiated by increasing dietary lipids. At this level, neuropeptides as ghrelin and POMC may represent potential modulators of inflammation based on their characteristic of being anti-inflammatory. Ghrelin can block the TLR4/MyD88/TRAF6/NF-KB pathway activated in microglia by SFA and decrease the activity of the AMPK and JUN, important for the production of IL-2. POMC derived peptides attenuate secretion of proinflammatory cytokines via MC4R-cAMP-PKA-CREB, which induces the release of IL-10. While MC1R receptor activation reduces the expression of the adhesion proteins E-selectin and VCAM, and reduce the activation of NF-kB in endothelial cells exposed to TNF-*α* (Figure 2). Overall, neuropeptides in the CNS modulate inflammation and migration of peripheral cells into the CNS via the BBB and may represent a molecular node during positive energy balance as is the obesity and maternal overnutrition.
